# Psychological Assessment of Infertile Females in Eastern Uttar Pradesh: A Cross-Sectional Evaluation

**DOI:** 10.7759/cureus.85862

**Published:** 2025-06-12

**Authors:** Sakshi Agarwal, Ananya Sharma, Lalit Kumar, Tej Bali Singh

**Affiliations:** 1 Department of Obstetrics and Gynecology, Institute of Medical Sciences, Banaras Hindu University, Varanasi, IND; 2 Department of Urology, Institute of Medical Sciences, Banaras Hindu University, Varanasi, IND; 3 Department of Preventive Medicine, Institute of Medical Sciences, Banaras Hindu University, Varanasi, IND

**Keywords:** anxiety, dass 21, depression, female infertility, stress, whoqol-bref

## Abstract

Infertility is not only a gynecological problem but also a psychological issue that holds significant social and cultural value for women. Women facing challenges in conceiving often struggle with emotional and psychological difficulties, including depression, anxiety, and stress. Infertile women are more likely to develop mental illnesses due to various etiological factors, which also impact their quality of life (QOL). This prospective study was conducted over six months, and 71 women between 20 and 40 years of age were interviewed. The primary objective of the study was to assess the impact of infertility on the mental health and QOL of women. Analysis was conducted using standardized tools: the Depression, Anxiety and Stress Scale - 21 Items (DASS-21) and WHO Quality of Life Brief Version (WHOQOL-BREF) questionnaires. Cronbach’s alpha was used to measure the reliability of the results. A p-value of <0.05 was considered statistically significant. The mean age of the infertile women was 29.2 ± 5.21 years. Using DASS-21, we found that patients experienced moderate to severe levels of depression, anxiety, and stress, with extremely severe anxiety being more prevalent. Rural infertile women were found to experience higher levels of stress compared to their urban counterparts. QOL appeared moderate, with social relationships being the most affected domain. Infertile women experience highly severe levels of anxiety and moderate to severe levels of depression and stress. These findings highlight the multidimensional impact of infertility and underscore the need for comprehensive healthcare approaches to address the psychological challenges faced by infertile women.

## Introduction

Infertility is a medical condition that makes it difficult or impossible for a couple to conceive after 12 months or more of regular and unprotected intercourse [1]. For women over 35 years of age, this period is considered to be 6 months. Infertility can occur due to dysfunction in either the male or female reproductive systems. However, females often suffer silently and to a greater extent compared to males, due to psychological, familial, and societal pressure. According to a 2023 report by the WHO, 1 in 6 couples, or 17.5% of adults globally, experience infertility at some point in their lives [2]. Infertility often causes overwhelming burdens stemming from physical, financial, interpersonal, and psychological stress [3]. According to the WHO, it is a global health issue of epidemic proportions and is a serious concern. The perception of quality of life (QOL), along with infertility-related depression, anxiety, and stress in affected females, needs to be analyzed to determine their impact on the condition and to develop strategies to help them cope with these emotional struggles.

Epidemiological studies have identified common causes of infertility in women, including obesity, menstrual disorders, diabetes, thyroid diseases, fallopian tube dysfunction, uterine abnormalities, and cervical issues [4]. According to the WHO, the overall prevalence of primary infertility in India ranges between 3.9% and 16.8% [5]. Approximately 8% of married women suffer from primary and secondary infertility, with 5.8% experiencing secondary infertility [6]. A study on infertility among Indian women found that its prevalence decreases with an increase in the woman’s age at the time of marriage [7]. Patients suffering from infertility often report feelings of anxiety, stress, depression, isolation, and loss of control. The levels of depression among couples experiencing infertility have been compared to those in cancer patients [8]. The inability to reproduce naturally may lead to feelings of guilt, shame, and low self-esteem, which in turn negatively impact depression, anxiety, stress levels, and overall QOL. Therefore, a psychological analysis of these factors in infertile females is necessary. The purpose of this study is to examine psychological findings and provide a clear interpretation of infertility-related psychological issues and their impact on QOL in affected women.

## Materials and methods

Ethics statement

The institute’s ethics committee (IEC/2024/7712) approved this study. The study included women who visited the Gynecology OPD, were diagnosed with infertility, and provided informed consent.

Study participants

This cross-sectional study was conducted in the Department of Obstetrics and Gynecology. Data were collected from 71 infertile women who visited between October 2024 and March 2025 for psychological assessment. Selection was done using convenience sampling. Patients were provided with a proforma consisting of general details designed by the researchers, including their names, religion, height, weight, age, etc., and were interviewed using the Depression, Anxiety and Stress Scale - 21 Items (DASS-21) and WHO Quality of Life Brief Version (WHOQOL-BREF) questionnaires, which were used for analysis.

DASS-21 questionnaire 

The Depression, Anxiety, and Stress Scale, abbreviated as DASS, is a widely used tool for psychological assessment. The original version consists of 42 items divided into three subscales: Depression, Anxiety, and Stress. It was developed by Lovibond and Lovibond in the 1990s. Its primary purpose is to support clinical diagnosis with psychometric indicators and to serve as a rapid and efficient measurement instrument for similar studies [9].

A shortened version, DASS-21, was later developed, encompassing all the dimensions of the original scale. Due to its simplicity, convenience, and broad applicability, DASS-21 has gained widespread acceptance. Since its introduction, various versions have been translated into different languages based on research requirements and practical considerations. In this study, the DASS-21 questionnaire consisted of 21 items across three self-reported scales designed to measure levels of Depression, Anxiety, and Stress. Each scale included seven items, graded on a 4-point Likert scale ranging from 0 to 3 (0: “Did not apply to me at all,” 3: “Applied to me very much or most of the time”). The scores from each scale were summed separately. As DASS-21 is the abbreviated version of DASS-42, each score was multiplied by 2 to calculate the final score. According to the DASS manual, the resulting scores were then graded as “normal,” “mild,” “moderate,” “severe,” or “extremely severe” [9-10].

WHOQOL-BREF questionnaire

Due to the negative impact of infertility on the QOL of affected females, the WHOQOL-BREF questionnaire was also administered. Infertile females answered both questionnaires (WHOQOL-BREF and DASS-21) in a single session. The original WHOQOL-100 is a comprehensive scale designed to assess various physical and psychological disorders. It was initially developed by the WHOQOL group as a 100-item form that allowed for a thorough evaluation of 24 aspects of QOL. However, the length of the questionnaire made it less feasible for routine use by researchers.

To address this, a shorter version, WHOQOL-BREF, was developed, which includes two questions on overall health and general QOL, along with one question from each of the 24 aspects to form a concise tool [11]. The items in this version are grouped into four domains: physical health (PH; 7 items), psychological well-being (PS; 6 items), social relationships (SR; 3 items), and environmental health (EH; 8 items). All questions are rated on a 5-point Likert scale (0-5). Raw scores for each domain were transformed to a 4-20 scale according to the manual, and then further converted to a 0-100 scale, where 100 indicates good QOL and 0 indicates poor QOL.

Statistical analysis

The collected proformas, including the questionnaires (DASS-21 and WHOQOL-BREF) and general data, were entered into Microsoft Excel. Statistical analysis was conducted using IBM SPSS Statistics software, version 28. Independent t-tests and one-way ANOVA were used to compare WHOQOL-BREF domain scores across demographic groups. The chi-square test of independence was applied to analyze categorical data from DASS-21 and demographic variables. A p-value of <0.05 was considered statistically significant. Cronbach’s alpha coefficient was used to assess the internal consistency of each WHOQOL-BREF domain and DASS-21 subscale, with a value of 0.7 or above considered acceptable [12].

## Results

Patient characteristics

A total of 71 women with infertility issues were included in this study. A proforma containing general details for each patient was completed, including various baseline characteristics (Table 1). The patients’ ages ranged from 20 to 40 years, with a mean age of 29.2 years (standard deviation: 5.21). More than half of the participants (60.6%) were graduates, 91.5% were unemployed, and approximately 81.7% lived in a joint family.

**Table 1:** Demographic characteristics of participants (n = 71). Age*: Mean = 29.2 years; SD = 5.21.

Variable	Category	Frequency (n)	Percentage (%)
Age (years)*	20-25	20	28.2
	26-30	21	29.6
	31-35	21	29.6
	36-40	9	12.7
Residence	Rural	39	54.9
	Urban	32	45.1
Religion	Hindu	64	90.1
	Muslim	7	9.9
Educational qualification	Graduate	43	60.6
	Intermediate	22	31
	Illiterate	6	8.5
Occupation	Employed	6	8.5
	Unemployed	65	91.5
Family type	Joint	58	81.7
	Nuclear	13	18.3

DASS-21 items 

Based on DASS-21 scores, all patients were categorized into varying levels of depression, anxiety, and stress, as shown in Table 2. Almost half of the patients were within the normal range for all three DASS subscales. Only 5.6% of participants were extremely severely depressed, and none had extremely severe stress. Anxiety had the highest prevalence of extremely severe cases (21.1%) among the three subscales.

**Table 2:** Distribution of DASS-21 scores. DASS-21: Depression, Anxiety and Stress Scale - 21 Items.

Score Category	Depression Level	Anxiety Level	Stress Level
Normal	39 (54.9%)	33 (46.5%)	48 (67.6%)
Mild	9 (12.7%)	4 (5.6%)	6 (8.5%)
Moderate	13 (18.3%)	14 (19.7%)	10 (14.1%)
Severe	6 (8.5%)	5 (7.0%)	7 (9.9%)
Extremely severe	4 (5.6%)	15 (21.1%)	–

A chi-square analysis of all the DASS subscales and selected demographic variables (age, residence, religion, education, occupation) revealed a significant association between residence and stress levels (p = 0.017), indicating that patients from rural areas experience more stress than those from urban areas (Table 3).

**Table 3:** Chi-square test results for the association between demographic variables and psychological distress. Note: * indicates statistically significant p-value (p < 0.05).

Variable Comparison	Chi-square Value	p-value
Age vs. Depression level	14.205	0.288
Age vs. Anxiety level	12.122	0.436
Age vs. Stress level	14.054	0.12
Residence vs. Depression level	4.18	0.382
Residence vs. Anxiety level	5.58	0.233
Residence vs. Stress level	10.226	0.017*
Religion vs. Depression level	4.641	0.326
Religion vs. Anxiety level	2.941	0.568
Religion vs. Stress level	4.612	0.203
Education vs. Depression level	10.104	0.258
Education vs. Anxiety level	7.933	0.44
Education vs. Stress level	9.789	0.134
Occupation vs. Depression level	3.417	0.491
Occupation vs. Anxiety level	1.341	0.854
Occupation vs. Stress level	0.894	0.827
Family type vs. Depression level	2.315	0.678
Family type vs. Anxiety level	3.891	0.421
Family type vs. Stress level	3.541	0.472

Evaluation of the DASS-21 subscale statistics provided insights into the psychological distress experienced by infertile women (Table 4). Depression had the highest mean score (22.31) compared to anxiety and stress, suggesting that depressive symptoms were more prevalent than anxiety or stress among infertile females. The p-values for all subscales were greater than 0.05 and thus were not statistically significant. Cronbach’s alpha values for all subscales were above 0.80, indicating high internal consistency and reliability of the DASS-21 questionnaire.

**Table 4:** DASS-21 subscale statistics. DASS-21: Depression, Anxiety and Stress Scale - 21 Items.

Subscale	Mean	SD	P-value	Cronbach’s Alpha
Depression	22.31	3.76	0.288	0.87
Anxiety	18.28	4.56	0.436	0.81
Stress	10.79	2.89	0.12	0.85

WHOQOL-BREF analysis

Independent t-tests were performed to compare WHOQOL-BREF domain scores across demographic variables, including current residence (urban vs. rural), religion (Hindu vs. Muslim), and occupational status (employed vs. unemployed). Only the comparisons based on residence yielded statistically significant findings. Environment domain scores differed significantly between urban and rural participants (p = 0.004), with urban women reporting a higher QOL (Table 5).

**Table 5:** WHOQOL-BREF t-test comparison between urban and rural groups. Group 1: Urban; Group 2: Rural Note: ** indicates statistically significant p-value (p < 0.05). WHOQOL-BREF: WHO Quality of Life Brief Version.

Groups Compared	WHOQOL-BREF Domain	Mean ± SD (Urban)	Mean ± SD (Rural)	t-value	P-value	Cronbach’s Alpha
Urban vs. Rural	Physical Health (D1)	23.75 ± 3.35	22.31 ± 3.76	1.51	0.136	0.76
Urban vs. Rural	Psychological Health (D2)	18.75 ± 4.47	18.28 ± 4.56	0.42	0.674	0.81
Urban vs. Rural	Social Relationships (D3)	10.97 ± 2.24	10.79 ± 2.89	0.29	0.773	0.68
Urban vs. Rural	Environment (D4)	29.06 ± 5.33	24.49 ± 7.48	3.02	0.004 **	0.85
Urban vs. Rural	Total WHOQOL Score	82.53 ± 12.30	75.87 ± 16.49	1.88	0.064	0.88

One-way ANOVA was conducted to determine whether significant differences existed in WHOQOL-BREF domain scores across variable groups (age, residence, religion, education, occupation). According to the analysis, none of the four domains showed statistically significant differences (p > 0.05), indicating that QOL does not significantly differ across groups within the infertile female population. These findings are presented in Table 6.

**Table 6:** One-way ANOVA results for WHOQOL-BREF domains. Note: Each domain refers to a specific aspect of quality of life. WHOQOL-BREF: WHO Quality of Life Brief Version.

Domain	Source	Sum of Squares	df	Mean Square	F-value	P-value
D1 (Physical Health)	Between Groups	14.984	3	4.99	0.36	0.77
	Within Groups	905.889	67	13.52		
	Total	920.873	70			
D2 (Psychological Health)	Between Groups	18.479	3	6.16	0.29	0.82
	Within Groups	1395.267	67	20.82		
	Total	1413.746	70			
D3 (Social Relationships)	Between Groups	25.785	3	8.59	1.28	0.28
	Within Groups	448.074	67	6.68		
	Total	473.859	70			
D4 (Environment)	Between Groups	8.932	3	2.97	0.05	0.98
	Within Groups	3362.645	67	50.18		
	Total	3371.577	70			

## Discussion

According to the WHO, approximately 60-80 million couples worldwide are infertile, and around 17.9 million couples face infertility in India [7]. Infertility is not only a medical condition but also associated with biopsychosocial issues that can lead to marital problems, social discomfort, sexual frustration, and psychological challenges such as depression, anxiety, and stress [13]. These psychological challenges are reported to be more prevalent in infertile women than in infertile men [14].

The mean age of infertile females in our study was 29.2 years. Similar findings were reported by Dyer SJ et al. and Bahadur A et al., who also observed comparable mean ages in their respective studies [15-16]. We interviewed 71 women facing infertility issues over a period of six months. The results present critical findings related to demographic factors, levels of psychological distress, associations between demographic variables and distress, and assessments of QOL across different domains.

According to the DASS-21 score distribution, a notable proportion of patients reported moderate to severe levels of depression, anxiety, and stress. Specifically, 21.1% of infertile patients were experiencing extremely severe anxiety, while 54.9% reported normal depression levels. Anxiety emerged as the most prevalent psychological concern among infertile females, suggesting that infertility may cause significant emotional suffering, particularly related to societal pressures, uncertainty about future conception, referrals to higher centers, or financial burdens. Alsahel M and Alghamdi M also reported cases of extremely severe anxiety in infertile women in a case study conducted in Saudi Arabia [17]. Furthermore, 67.6% of the participants did not report significant stress levels, suggesting that stress may not be as consistently experienced as anxiety and depression. These findings imply that while infertility may not always manifest as stress, it does contribute significantly to anxiety regarding treatment outcomes and future possibilities.

Chi-square analysis examining associations between demographic factors and psychological issues revealed that most selected variables, including age, education, and occupation, did not show statistically significant associations with depression, anxiety, or stress. However, the variable of residence was significantly associated with stress (p = 0.017), indicating that stress levels may vary between urban and rural women. This difference could be attributed to disparities in healthcare accessibility, cultural expectations, or social support systems related to infertility.

The mean scores for depression (22.31 ± 3.76), anxiety (18.28 ± 4.56), and stress (10.79 ± 2.89) highlight the substantial psychological burden experienced by the participants. High Cronbach’s alpha values (ranging from 0.81 to 0.87) indicate that the DASS-21 scale demonstrated strong reliability and internal consistency in this population. Based on WHOQOL-BREF scores across its four domains, physical health (D1), psychological health (D2), social relationships (D3), and environment (D4), the social relationships domain had the lowest mean value (10.87 ± 2.60), suggesting that infertile females face considerable challenges in personal relationships. This may be due to a lack of spousal support, cultural stigma, or marital strain caused by childlessness. In contrast, a study by Bakhtiyar K et al. found higher scores in the social domain among infertile women in Iran, which may be attributed to greater emotional support from family in Iranian culture [18]. The highest mean score in our study was observed in the environmental domain (26.28 ± 7.10), indicating relatively stable access to resources and healthcare among participants.

Differences in WHOQOL-BREF scores across different groups were analyzed using one-way ANOVA; the results did not indicate significant differences across domains (p > 0.05). This suggests that regardless of age, occupation, or education level, infertile females report similar QOL across all four domains. Similarly, a QOL analysis of infertile women in Bangladesh by Ishrat S et al. found moderate levels of well-being [19].

These findings indicate that psychological problems, particularly anxiety, are persistent among infertile females. The lack of significant associations between DASS scores and most demographic factors, except for stress and residence, shows that infertility-related psychological distress is experienced broadly across different groups. This highlights the need for mental health support interventions that address psychological and emotional well-being irrespective of demographic variations. The WHOQOL-BREF results also revealed that infertility primarily affects social relationships, likely due to societal pressure and interpersonal strain.

The strength of this study lies in its inclusion of a diverse age range (20-40 years), allowing for a comprehensive analysis across different life stages. Additionally, the use of validated tools such as the DASS-21 and WHOQOL-BREF questionnaires lends credibility to the results. A rigorous statistical analysis was conducted using the latest version of SPSS, reinforcing the study's reliability. However, there are a few limitations: the sample size was relatively small; the study period was limited to six months; and comparisons between fertile and infertile females were not included. Moreover, the study did not delve deeply into the etiological factors of infertility.

## Conclusions

Our results revealed that infertile females in rural areas exhibited higher levels of anxiety. Overall, depression appeared to be elevated among all infertile patients, regardless of demographic factors. The QOL across the cohort was moderate, with the greatest impact observed in the domain of social relationships. These findings highlight that psychological well-being is a critical concern in this population. Based on these results, there is a pressing need for psychological counseling, targeted interventions addressing depression and anxiety, and peer support programs. Such measures may contribute to improved mental health and enhanced social functioning among infertile women.
